# Retracted: Modulation of Signal Proteins: A Plausible Mechanism to Explain How a Potentized Drug Secale Cor 30C Diluted beyond Avogadro's Limit Combats Skin Papilloma in Mice

**DOI:** 10.1155/2021/2108674

**Published:** 2021-01-15

**Authors:** Evidence-Based Complementary and Alternative Medicine

*Evidence-Based Complementary and Alternative Medicine* has retracted the article titled “Modulation of Signal Proteins: A Plausible Mechanism to Explain How a Potentized Drug Secale Cor 30C Diluted beyond Avogadro's Limit Combats Skin Papilloma in Mice” [1], due to concerns with the figures as originally raised on PubPeer [2].

Overlap was identified between Figures 3(d), 3(e), and 3(f) and between Figures 3(a) and 3(b). The authors explained that some overlapping features are to be expected and also provided alternative images. Due to the high level of overlap, this explanation was not determined to be satisfactory and the article is therefore being retracted with the agreement of the editorial board. The authors do not agree to the retraction.
